# Toward reanimating the laughter‐involved large‐scale brain networks to alleviate affective symptoms

**DOI:** 10.1002/brb3.2640

**Published:** 2022-06-10

**Authors:** Shahab A. Zarei, Seyedeh‐Saeedeh Yahyavi, Iman Salehi, Milad Kazemiha, Ali‐Mohammad Kamali, Mohammad Nami

**Affiliations:** ^1^ Institute of Neuroscience The Center of Excellence in Brain and Intelligence Technology State Key Laboratory of Neuroscience Key Laboratory of Primate Neurobiology Chinese Academy of Sciences Shanghai China; ^2^ Department of Neuroscience School of Advanced Medical Sciences and Technologies Shiraz University of Medical Sciences Shiraz Iran; ^3^ DANA Brain Health Institute Iranian Neuroscience Society Fars Chapter Shiraz Iran; ^4^ Neuroscience Laboratory (Brain, Cognition and Behavior) Department of Neuroscience School of Advanced Medical Sciences and Technologies Shiraz University of Medical Sciences Shiraz Iran; ^5^ Society for Brain Mapping and Therapeutics (SBMT) Los Angeles California USA; ^6^ Harvard Alumni for Mental Health Middle‐East Ambassador Dubai UAE

**Keywords:** eye‐tracker, face analysis, laughter network, polygraphy, qEEG

## Abstract

**Introduction:**

The practicality of the idea whether the laughter‐involved large‐scale brain networks can be stimulated to remediate affective symptoms, namely depression, has remained elusive.

**Methods:**

In this study, 25 healthy individuals were tested through 21‐channel quantitative electroencephalography (qEEG) setup upon resting state and while submitted to standardized funny video clips (corated by two behavioral neuroscientists and a verified expert comedian, into neutral and mildly to highly funny). We evaluated the individuals’ facial expressions against the valence and intensity of each stimulus through the Nuldos face analysis software. The study also employed an eye‐tracking setup to examine fixations, gaze, and saccadic movements upon each task. In addition, changes in polygraphic parameters were monitored upon resting state and exposure to clips using the 4‐channel Nexus polygraphy setup.

**Results:**

The happy facial expression analysis, as a function of rated funny clips, showed a significant difference against neutral videos (*p* < 0.001). In terms of the polygraphic changes, heart rate variability and the trapezius muscle surface electromyography measures were significantly higher upon exposure to funny vs. neutral videos (*p* < 0.5). The average pupil size and fixation drifts were significantly higher and lower, respectively, upon exposure to funny videos (*p* < 0.01). The qEEG data revealed the highest current source density (CSD) for the alpha frequency band localized in the left frontotemporal network (FTN) upon exposure to funny clips. Additionally, left FTN acquired the highest value for theta coherence *z*‐score, while the beta CSD predominantly fell upon the salience network (SN).

**Conclusions:**

These preliminary data support the notion that left FTN may be targeted as a cortical hub for noninvasive neuromodulation as a single or adjunct therapy in remediating affective disorders in the clinical setting. Further studies are needed to test the hypotheses derived from the present report.

## Graphical abstract

The qEEG data revealed the highest current source density (CSD) for the alpha frequency band localized in the left frontotemporal network (FTN) upon exposure to funny clips. Additionally, left FTN acquired the highest value for theta coherence *z*‐score, and the beta CSD predominantly fell upon the salience network (SN). These preliminary data support the notion that left FTN may be targeted as a cortical hub for noninvasive neuromodulation as a single or adjuvant therapy for affective disorders in the clinical setting.

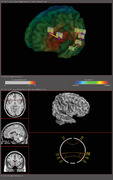

## 1 INTRODUCTION

While laughter is among the most widespread human behaviors, little research has scrutinized its neuroscience underpinnings (Martin, 2002; Savage et al., 2017). If the neural dynamics of laughter are better explained, we may expect novel avenues to potentially help affective disorders, namely depression, through reanimating laughter‐related brain networks. That said, the existing evidence on these facts appear to be thin. Compared to an almost 7700 retrievable scientific papers in our PubMed search using the MeSH (Medical Subject Headings) terms “emotion,” “fear,” and “expression” (Jan 2017–Jan 2021), only 210 papers were extracted when the search terms changed into “emotion,” “expression,” and “laughter.” Furthermore, from the relevant research papers retrieved on laughter, only a handful have experimentally studied this phenomenon (Farkas et al., 2021, Jáuregui & Lecoq; Martinelli et al., 2021; Yang et al., 2021).

Informed by the existing research, laughter is largely considered as a social behavior. As such, sociocultural variables seem to play a key part in deriving our mirth (Jáuregui & Lecoq, Wood, 2020). While we tend to laugh mostly at jokes or humor, genuine laughter seems to frequently occur during social encounters and conversations. In other words, some comments or statements that are not humorous on their own may simply elicit laughter in a conversation. In that sense, often a genuine smile and laughter indicate a reaction to shared interests, affirmation, and social affiliation (Ginzburg et al., 2020; Mazzocconi et al., 2020).

Given the paucity of existing evidence on the neuroscience of laughter, research needs to experimentally address key questions such as: (1) Are the presumptive laughter networks (i.e., the connectome between laughter‐involved cortical and subcortical brain regions) cross‐culturally different? (2) How does laughter relate to the socioeconomic, cultural, and linguistic differences in various populations and age groups? and (3) What are the neurodynamics of large‐scale brain networks in laughter? Moving forward, the impact of modulating laughter‐involved neural networks using noninvasive brain stimulation modalities can be an interesting idea to explore. Future hypotheses may consider the effectiveness of noninvasive transcranial electrical stimulation in modulating the cortical hotspots involved in laughter aiming to possibly alleviate symptoms in affective dysregulations, namely depression.

### 1.1 What does the science of laughter imply in affective neuroscience?

The existing reports on the neural substrates of laughter lack consensus. One justification could be the complexity of laughter as a socio‐behavioral, cognitive, emotional, and cultural phenomenon. It has been accepted that the stereotyped responses in laughter are mediated by subcortical structures, especially the hypothalamus, but cerebral cortex can potentially modulate them. Therefore, it can be hypothesized that the activation and inhibition of the involved networks within the cerebral cortex potentially reinforce the act of laughter or the sense of mirth (Caruana et al., 2015; Farkas et al., 2021; Martinelli et al., 2021; Mazzocconi et al., 2020).

Laughter involves both emotional and nonemotional/motor neurobehavioral components. Earlier research works tested the hypothesis that each component of laughter is separately codified by a distinct large‐scale cortical network. For example, a multifiber tractography study revealed that not only the known cortical and subcortical regions, but also primary motor cortex extended descending projections upon laughter (Gerbella et al., 2021).

In a comprehensive neuroimaging study, an evoked regional brain activity was ubiquitously reported when individuals were submitted to humorous vs. neutral cues. The investigation revealed that exposure to complex humor provokes the regional cerebral blood flow and blood oxygen‐dependent signals in bilateral amygdala and inferior frontal gyri, which are linked to emotional processes. Additionally, cortical areas involved in language, semantic knowledge, and theory of mind similarly demonstrate a surge in activity when individuals are exposed to written and visual humor cues (Farkas et al., 2021).

Given these facts , we hypothesize that modulating the cortical and subcortical hotspots involved in laughter and humor perception is a potential corridor connecting laughter neuroscience to future solutions in affective disorders. We also hypothesize that the presumptive laughter networks are cross‐culturally different given the socioeconomic, cultural, and linguistic disparities in various populations and age groups.

Though laughter generally corresponds to joy and happiness, its threshold and dynamics might differ among languages and cultures (Farkas et al., 2021). In other words, while laughter is identifiable as a universal phenomenon across cultures, its triggers (e.g., verbal, visual, or semantic motives) seem varied from one culture to another. As such, exploring the substrates of cultural variability in humor and subsequent laughter is an intriguing area of research. In that vein, cognitive processing, social norms and definitions, and language‐related variable may at least partly explain the existing heterogeneity in cultural aspects of laughter (Jáuregui & Lecoq).

Adding to the factors described earlier (language and culture), several other variables such as age, gender, and education can potentially affect laughter on a contextual basis. Moreover, other confounders, such as the subject's phase of the menstrual cycle, handedness, educational status, and differences in language and culture are expected to affect the phenomenon (Jáuregui & Lecoq). Therefore, identifying the effects of such factors would help designing possible laughter interventions for different study populations and settings.

### 1.2 The rationale behind the present research

There are some empirical study findings on the neurodynamic changes in brain circuitries following laughter, but the interplay between large‐scale cortical networks in laughter has by far remained unexplained. Therefore, one might be prompted to design an investigation to examine the neural networks involved in laughter based on which laughter networks can be reanimated to alleviate affective symptoms. Over and above, future works would also need to formulate neurofunctional models in humor perception and laughter through social neuroscience perspectives.

There are two primary aims of our study: (1) To investigate the central and autonomic nervous system signatures upon the induction of genuine laughter and (2) to ascertain which large‐scale brain network(s) governs such dynamics. We determined these earlier‐mentioned aims through concurrent qEEG and polygraphy cross‐validated with eye‐tracking while subjects were submitted to the laughter tasks.

## 2 MATERIALS AND METHODS

The study was carried out according to the ethical guidelines laid down by Shiraz University of Medical Sciences (SUMS) and in line with the declaration of Helsinki and its later amendments. Participants were briefed about the protocol and provided written informed consent. Prior to commencing the study, ethical clearance was sought from the SUMS IRB committee.

### 2.1 Participants’ inclusion/exclusion criteria

Here we tested 25 healthy (based on an interview), right‐handed college students (age: 19–30, 10 male and 15 female) without any history of neuropsychological problems or current use of psychotropic medications. The population was normalized in terms of personality traits (using the MMPI—Minnesota Multiphasic Personality Inventory), and their experience of watching stand‐up comedy (visual analog scale).

### 2.2 Quantitative Electroencephalography

A 21‐channel EEG device (EEG 3840, Mitsar 201) was used to evaluate the effect of funny clips on the EEG background. Electrodes were placed according to the International 10/20 System (average referenced). The impedance beneath each electrode was kept <5 kΩ and the sampling rate upon data collection was 250 samples per second. The Neuroguide software (v.3.0.2 2001–2018 Applied Neuroscience Inc., USA) was employed for signal processing as well as removal of any muscle‐ or eye movement artifacts (sensitivity was set at high level and *z*‐score threshold was 2) as well as the quantitative data analyses.

### 2.3 Polygraphy

**FIGURE 1:**
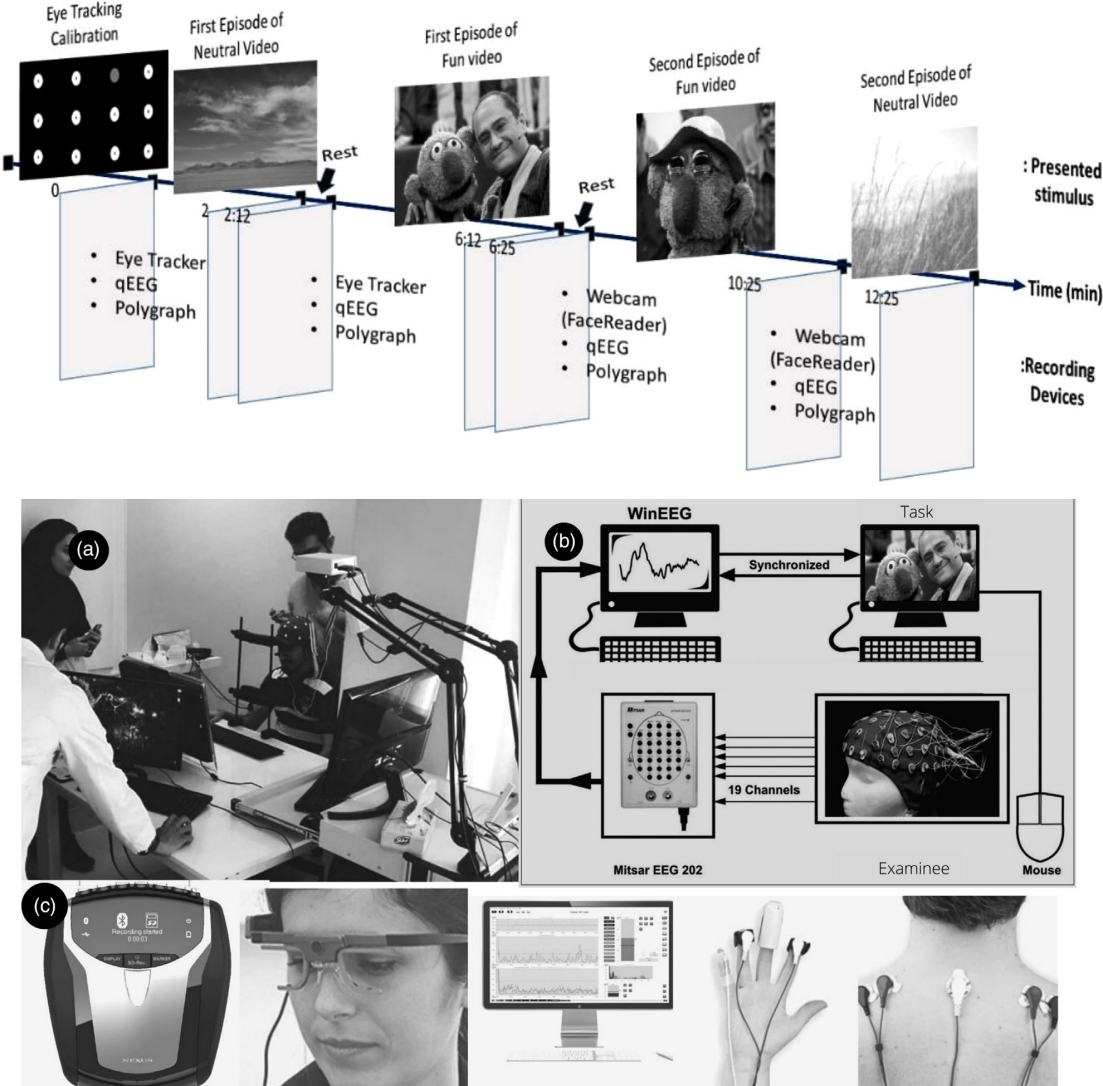
Upper panel: Study protocol, timeline of presented stimulus and active recording devices throughout the experiment. Prior to the experiment, calibration procedure was performed using a 12‐point calibration. As the initial part of the experiment, a neutral video content was displayed on a monitor, placed 60 centimeters from the examinee. This session was followed by the presentation of the funny content, which lasted for 4 min, after a short 2‐min break. During these two initial states, data from eye‐tracker, qEEG, and Polygraph were recorded synchronously. Then, the experiment proceeds by another 2‐min rest session, which ended by the presentation of another funny content and neutral one, while they lasted for 4 and 2 min, respectively. Meanwhile, from the beginning of second funny clip, the experiment was conducted by recording the participant's facial emotion reaction, utilizing face reader, and qEEG and Polygraphy data. Lower Panel: Data acquisition setup. (a) the experimental platform including simultaneous qEEG, polygraphy, face reader, eye‐tracking, and synchronized task submission setup. (b) the task‐concurrent qEEG acquisition setup including the EEG device, electrocap, 2 PCs, a mouse, and the WinEEG software. (c) (left to right) the Nexus‐10 polygraphic device, eye‐tracking setup, polygraphy recording, sensor placement, and trapezius muscle surface electromyography (EMG) electrodes’ position.

**FIGURE 2:**
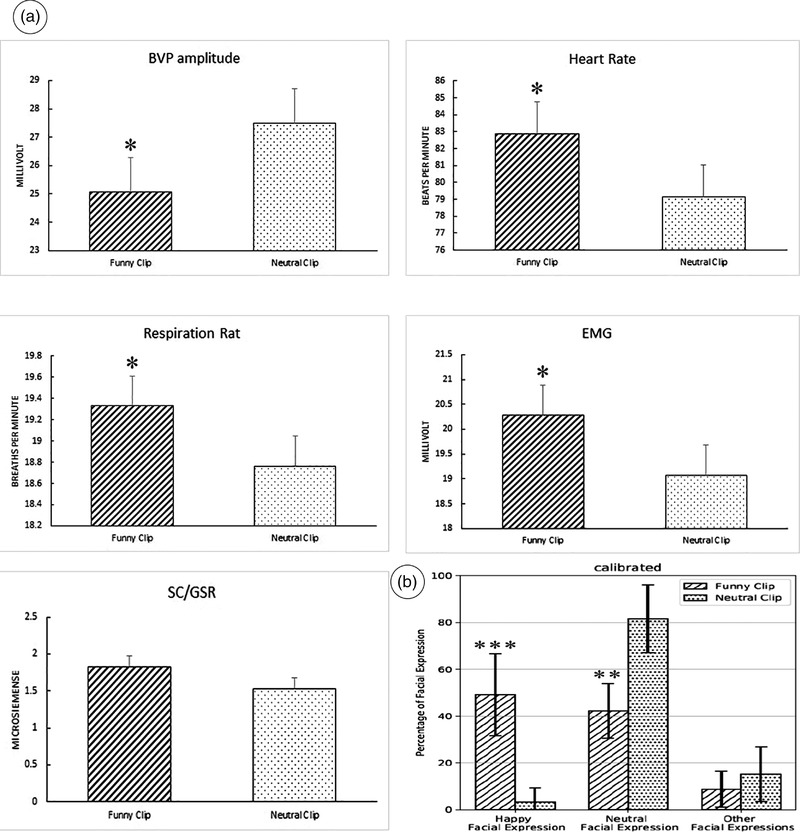
(a) Using a polygraph device, changes in polygraph parameters, including changes in heart rate and heart rate variability (HRV), blood volume pulse (BVP), respiration rate, electromyography (EMG), and skin conductance (SC) while watching neutral videos versus funny clips were reviewed. A notable comparative increase in heart rate, EMG (surface EMG over the trapezius muscles as shown in Figure 1), SC, and respiratory rate as well as a decreased BVP when subjects were submitted to funny clips (the act of genuine laughter as verified by face reading) suggest the sympathetic overdrive. Vertical bars indicate standard deviation of the means. *p < 0.05. (b) Face reader software results. The calibrated facial expression output is illustrated in the graph [happy facial expression (***p‐value: 0.0003); neutral facial expression (**p‐value: 0.001); other facial expression (*p*‐value = 0.11)]. Vertical bars indicate standard deviation of the means.

**FIGURE 3:**
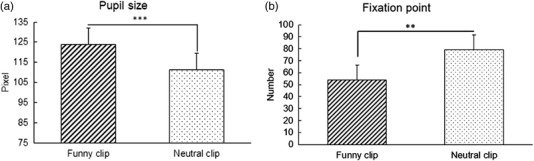
(a) The average pupil size while watching funny and neutral clips. (b) The average number of fixation points per person while watching funny and neutral clips. Vertical bars indicate standard deviation of the means. ***p‐value: 0.0001 **p‐value: 0.002.

**FIGURE 4:**
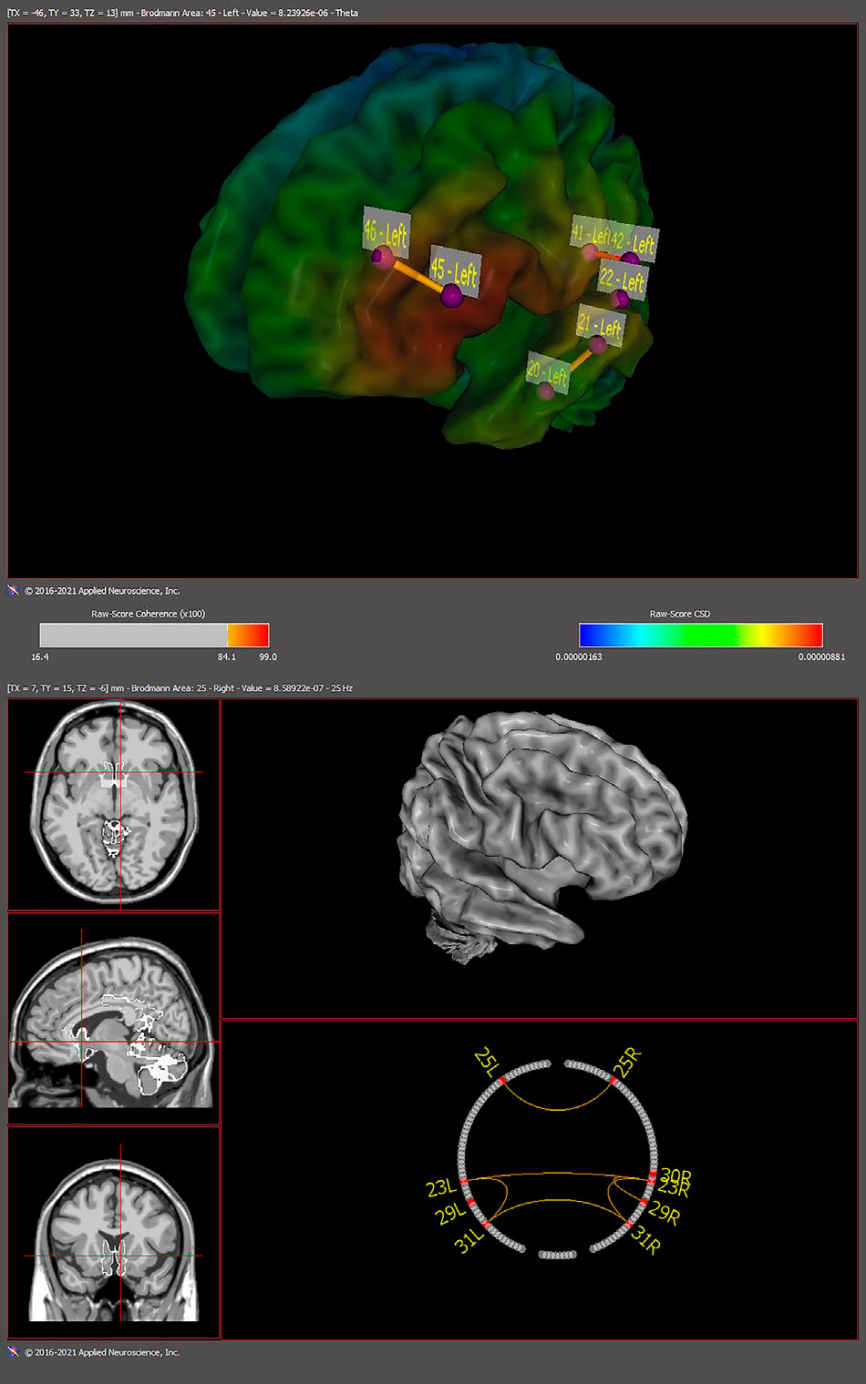
Left frontotemporal theta coherence network during funny clips. Raw‐Score coherence: 84–99 (100X). The right frontotemporal network demonstrated the highest value for alpha current source density (CSD) gain (Brodmann area: 22, Value: 4.482e‐06). The salience network acquired the highest beta center values in the right Caudate (Brodmann area: 25, Value: 8.58922e‐07).

**FIGURE 5:**
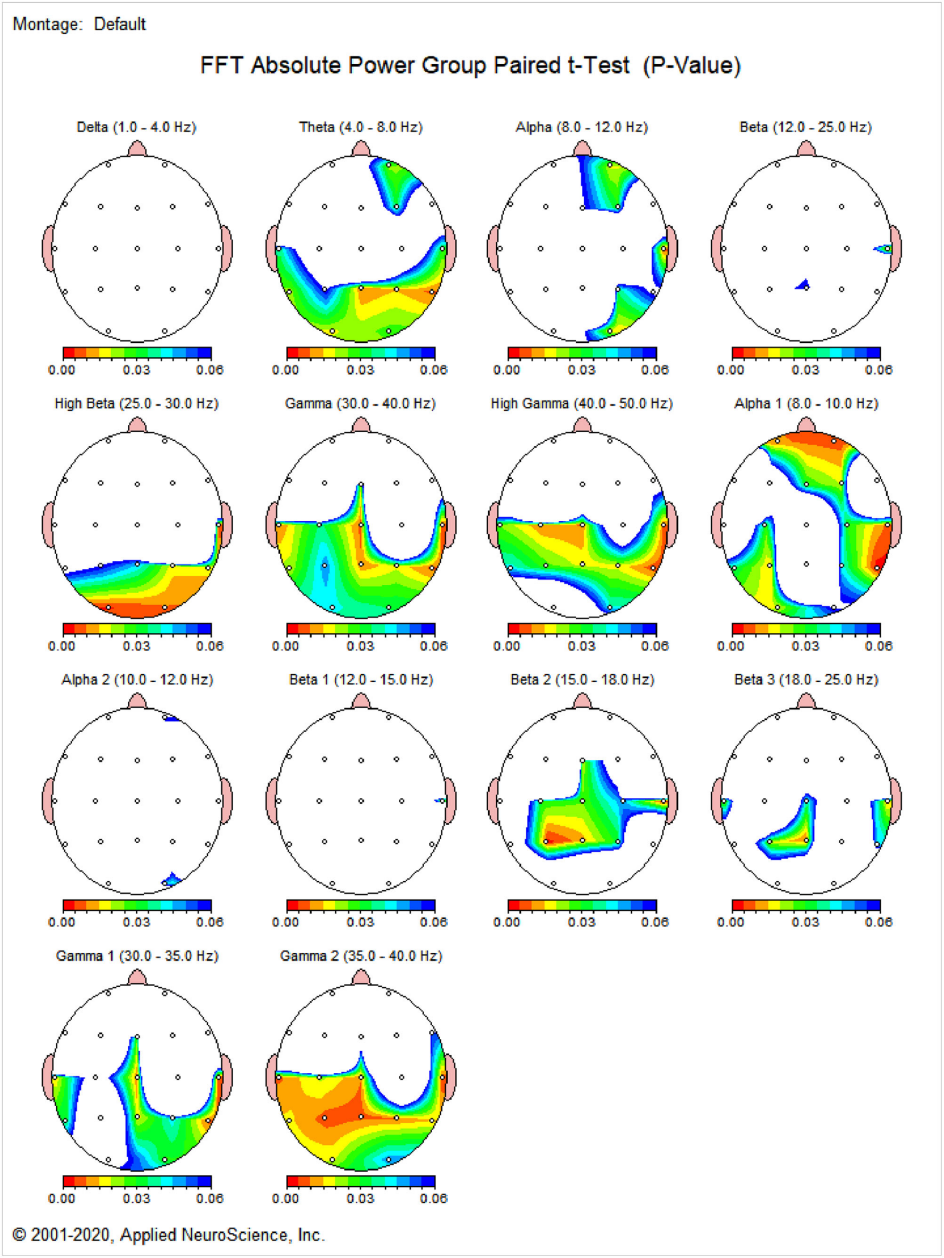
Absolute power paired *t*‐Test (*p*‐Value). Paired *t*‐test was used to evaluate any significant differences in the absolute power gain upon participants’ submission of the funny and neutral video clips.

Polygraphic data were acquired using the NeXus‐10 setup/sensors and Biotrace+ software (www.mindmedia.com). The Nexus polygraph device measured changes in heart rate variability (HVR), blood volume pulse (BVP), respiration rate (RR), surface electromyography (EMG), and skin conductance (SC) while individuals were at resting state or submitted to the funny and neutral video clips. The Nexus‐10 polygraphy device and its electrodes’ position are briefly illustrated in Figure 1 (see Figures 2, 3, 4, 5).

### 2.4 Eye‐tracker

Using an eye‐tracker, the pupil size of both eyes and saccadic eye movement (gaze plot) were monitored while subjects were watching the funny and neutral clips (2 min each). Also, the number of fixation points was recorded during the same period. The pupil size was obtained from the average pupil size of the right and the left eye over the task period.

The eye‐tracker setup evaluated the effect of funny clips on saccades and pupil diameter. Using the APRA head‐mounted binocular eye‐tracking system (Tehran, Iran, apra‐tech.com/eyetracker), eye gaze data were sampled at 120 Hz. A 12‐point board calibration was performed before submitting the participants to the experimental blocks. The subjects' eye gaze data were continuously monitored thorough the initial step of the experiment and data were recorded using the APRA gaze tracker software. Upon conclusion, the output data were processed using the APRA gaze analyzer for each trial to define the fixation plot and pupil diameter. Results were categorized into different types of eye movements such as fixations, saccades and, finally, were stored in a .csv (format) file to be statistically processed later in the analysis.

### 2.5 Face reading assessment

According to the Ekman's Emotion Model (Wang et al., 2020), basic emotional expressions (including, happy, sad, angry, surprised, scared, disgusted and a neutral state) were detected using Face reader software version 8.0 (FR, Noldus, 2019). Indeed, the software (trained to register activation of 20 action units, i.e., 1, 2, 4, 5, 6, 7, 9, 10, 12, 14, 15, 17, 20, 23, 24, 25, 26, 27, and 43) calculates the intensity of positive emotion (happiness or surprise) minus the negative emotion with the highest intensity indicated as an index of emotional valance. The score for valance suggests whether the patient feels in a pleasant or unpleasant way, ranging from −1 (unpleasant facial expression) to 1 (pleasant facial expression). Another output, arousal, represents to what extent the participant is emotionally aroused (+1) or not (−1). With no compromise over the accuracy, great convergent validity was reported for this software in accordance with the manually coded facial action coding system (FACS) ratings. FR measurements were calibrated as recommended by the software manual (Den Uyl & Van Kuilenburg, 2005; Küntzler et al., 2021; Sarkol‐Teulings, 2021).

### 2.6 Experimental design

This has been a single‐arm observational study and the data were collected over a single session. The experiment was carried out in a quiet room with controlled level of luminance. Prior to the experiments, consent forms were signed. Participants were asked to sit on a comfortable chair before a monitor display (distance of 40 cm from the 17″ monitor display with 1024768 pixels resolution). After cleaning the right‐hand finger skin using the abrasive gels, polygraphy sensors (BVP [blood volume pulse], skin conductance, and temperature sensor) were placed over the fingers, respiration sensor was placed around the abdomen, and EMG sensors were fixed over the upper part of trapezious muscles. After donning the EEG cap, participants were asked to immobilize their heads using a chinrest while the eye‐tracker setup was being fixed and calibrated. Later, the participants were asked to watch the clip containing two blocks of funny and two blocks of neutral parts while having their EEG, polygraphy, and eye‐tracking data recorded. When the first series of neutral and emotional video contents were displayed, the eye‐tracker was removed from the setup to have a precise analysis using face reader in the following steps of the experiment. We used a mouse button trigger maker to mark the EEG tracing synchronously with the tasks (WinEEG Software). As such, the time points at which the stimuli were delivered during the data acquisition process were concurrently marked throughout the recording (Jindrová et al., 2020) (See Figure 1).

## 3 RESULTS

### 3.1 The polygraphic and face reading analyses

Our polygraphic results showed a significant difference between the EMG response during the funny and neutral clips. The same was for neutral clips. Likewise, HRV changes showed a significant increase (*p* < 0.0001) upon watching funny clips.

Turning into the face reading experiment, when participants watched the funny clips, the proportion of happy facial expression was significantly (*p* = 0.0003) higher than that of neutral clips. Conversely, neutral facial expression demonstrated a significant increase (*p* = 0.001) when the participants were watching the neutral clips. No significant changes (*p* = 0.11) were noted in other facial expressions upon watching either types of clips.

### 3.2 Eye‐tracking analysis

Our findings demonstrated a significant difference between the pupil size for the funny and neutral clips favoring a wider pupil size for funny video exposures (*p* < 0.0001). The average number of fixation points while watching funny clips showed a significant difference to the neutral clips. There was a significant increase (*p* = 0.0028) in the number of fixation points while subjects were submitted to the neutral clips.

### 3.3 Quantitative Electroencephalography

The frontotemporal network (FTN) theta coherence *z*‐score was found to gain when subjects were watching the funny clips. Also, the current source density (CSD) for alpha band represented the highest gain in the right inferior frontal region (Brodmann area 22) when subjects were watching the funny clips. In addition, the highest beta center values were recorded within the salience network (SN) (including the right caudate). Paired *t*‐test analysis also revealed significant differences in absolute power between the states where the participants were submitted to the funny and neutral videos (*p* < 0.05).

## 4 DISCUSSIONS

Our study empirically assessed the neural correlates of genuine laughter behavior using the qEEG and polygraphic data. Individuals’ responses to funny vs. neutral clips were verified using the face reader and eye‐tracking setups. Our findings indicated significant changes in the autonomic nervous system dynamics (including as well as a qEEG‐informed notably enhanced spectral distribution of theta and alpha frequency bands in the left frontotemporal and left inferior frontal areas upon laughter).

While this has not been already tested from the brain mapping perspective, laughter is anecdotally known to alleviate symptoms of anxiety, depression, and affective ailments. There seems to be no succinct “laughter center” in the brain; instead, the phenomenon involves an interplay between the large‐scale cortical and subcortical networks (Farkas et al., 2021; Leow et al., 2016).

Laughter is usually a physical expression of merriment and pleasure (such as joy, mirth, happiness, relief, etc.) as a patterned motor response, that is, an inarticulate vocalization. The phenomenon often signals acceptance and positive interactions with others. Arguably, laughter is considered to be contagious, and one's act of laughter can reciprocate others’ as a positive feedback (Camazine et al., 2020).

In addition to its social benefits, the psychological effects of laughter can increase mood, optimism, energy, and cognitive function, potentially lessening anxiety, stress, loneliness, depression, and emotional tension (Mora‐Ripoll, 2011). Interestingly, the physiological benefits of laughter have also been reflected not only on blood pressure, cardiovascular and respiration system; but also on relieving pain, relaxing muscles, and improving cognitive and immune systems (Rodden, 2018).

Other than these benefits , the importance of humor as a coping strategy in caring for older patients, particularly those with cognitive impairment (Liptak et al., 2014), dementia (Takeda et al., 2010), or depression (Konradt et al., 2013) is articulated in the literature. For instance, it was shown that participation in a humor‐therapy group significantly reduced the use of psychotropic drugs (antipsychotics) in nursing homes (Leow et al., 2016). Moreover, older adults taking part in a humor therapy program showed less chronic pain and more self‐reported happiness compared to a control group (Tse et al., 2010).

With respect to the latest imaging studies in normal subjects, many cerebral areas are proposed to be associated with the processing of laughter. Such areas may retain excitatory or inhibitory properties toward laughter. As such, some have been targeted for the treatment‐resistant depression. For instance, the subcallosal anterior cingulate cortex (ACC) has been shown to be involved in negatively charged memories (Lozano et al., 2008).

Strictly speaking, the expression of laughter seems to be controlled mainly by two interacting networks (Lauterbach et al., 2013). The involuntary, emotionally driven system might be responsible for expressive laughter and should involve cortical and subcortical brain regions. Namely, the ACC, insula, mesial temporal, and or orbito‐frontal cortices, as well as subcortical structures including the amygdala, hypothalamus, subthalamic areas, the periaqueductal gray, and the dorsal/tegmental brainstem are known to be key substrates of the network. In contrast, the voluntary system could be controlled by the pre‐supplementary motor area (SMA)/SMA and frontal motor opercular areas ending up to the motor cortex and pyramidal tract to the ventral brainstem (10). Furthermore, the laughter response is governed by a laughter‐coordinating center in the dorsal upper pons when “laughter without emotional content” is produced (Wild et al., 2003).

In line with what we demonstrated here, some researchers have shown that laughter networks and language processing areas are interdigitated in one's dominant hemisphere (Vaca et al., 2011; Yamao et al., 2015).

## 5 CONCLUSION

The present results put forward some possible corridors through which laughter‐involved cortical brain areas can be modulated. Identifying such cortical hubs, pathways, and the related key network dynamics would pave the path for further studies to alleviate affective dysregulations, including depression, generalized anxiety, post‐traumatic stress disorder, and related conditions potentially through diminishing the laughter threshold by stimulating key cortical hubs or networks.

## CONFLICT OF INTEREST

Authors have no conflict of interest to disclose with regard to this manuscript.

### PEER REVIEW

The peer review history for this article is available at https://publons.com/publon/10.1002/brb3.2640

## Data Availability

The datasets used and/or analyzed during the current study are available from the corresponding author upon reasonable request.
